# Editorial: Plant diversification driven by genome and chromosome evolution and its reproductive and environmental correlates

**DOI:** 10.3389/fpls.2025.1665681

**Published:** 2025-09-29

**Authors:** Carmen Benítez-Benítez, Angelino Carta, Marcial Escudero, Enrique Maguilla, Santiago Martín-Bravo

**Affiliations:** ^1^ Department of Plant Biology and Ecology, Faculty of Biology, University of Seville, Seville, Spain; ^2^ Department of Biology, Botany Unit, University of Pisa, Pisa, Italy; ^3^ Botany Area, Department of Molecular Biology and Biochemical Engineering, Universidad Pablo de Olavide, Seville, Spain

**Keywords:** chromosome, microevolution, macroevolution, genome, biodiversity, diversification

The intricate processes of plant diversification are profoundly influenced by changes at the genomic and chromosomal levels (Wang et al., 2025), which in turn impact reproductive strategies and adaptation to diverse environments (Hansen et al., 2012; Bragg et al., 2015). This Research Topic, “Plant Diversification Driven by Genome and Chromosome Evolution and Its Reproductive and Environmental Correlates,” brings together a collection of nine insightful articles that explore these multifaceted interactions across a diverse range of plant lineages. From ancient polyploidization events to contemporary local adaptations, these articles highlight the dynamic interplay between genomic architecture, chromosomal rearrangements, and evolutionary success in the plant kingdom.

Several articles in this Research Topic shed light on the fundamental role of genome and chromosome evolution in shaping plant diversity. Gallego-Narbón et al. investigated the influence of whole-genome duplication (WGD) events on the evolution of the ginseng family (Araliaceae). Their phylogenomic analyses suggest that ancient hybridization and WGDs preceded the origin and diversification of major clades within the family, underscoring the long-term impact of these events (Soltis and Soltis, 2016). Building on this theme, Benítez-Benítez et al. provided a comprehensive review bridging micro and macroevolutionary processes through chromosomal dynamics. The reviewed evidence reflects that while polyploidy and dysploidy are known drivers of speciation, other chromosomal rearrangements like insertions, deletions, inversions, and translocations are increasingly recognized for their role in local adaptation and speciation. In addition, it suggests that certain chromosomal dynamics become fixed over macroevolutionary time after the filter of speciation (Rolland et al., 2023).

Further exploring the impact of polyploidy, Sharovikj Ivanova et al. employed an integrative taxonomic approach to uncover cryptic diversity within the *Euphorbia nicaeensis* alliance (Euphorbiaceae). Their research revealed multiple polyploidization events and complex phylogenetic patterns, resulting in the description of new species and emphasizing the significant role of polyploidy driving diversification within this group. The dynamic behavior of repetitive DNA elements in polyploid genomes is addressed by Decena et al. in their study of *Brachypodium* grasses (Poaceae). They demonstrate how the expansion and contraction of repeat elements contribute to genome size variation and respond to the “polyploid genome shock hypothesis,” revealing contrasting evolutionary outcomes across different *Brachypodium* lineages. Meanwhile, Beránková et al. uncover striking variations in chromosome structure within *Musa acuminata* subspecies (Musaceae) and cultivars, underscoring the profound impact of hybridization and polyploidization on chromosomal rearrangements in cultivated bananas.

Beyond the direct impact of genome and chromosome evolution (Lucek et al., 2023; Mohan et al., 2024), this Research Topic also delves into how these changes correlate with reproductive traits and environmental adaptation. Valdés-Florido et al. investigated the interplay between climatic niche evolution, polyploidy, and reproductive traits in the Mediterranean genus *Centaurium* (L.) Hill. Their findings suggest that polyploidization is a crucial process for plant evolution in the Mediterranean region (Escudero et al., 2018), facilitating speciation and diversification into new areas with different climates, and involving shifts in climatic niches and the evolution of novel reproductive strategies. This emphasizes the adaptive advantage conferred by genomic changes in response to environmental pressures (Hansen et al., 2012).

The genetic basis of local adaptation to environmental challenges is further investigated by Zou et al. in the cold-tolerant mangrove *Kandelia obovata* Sheue, Liu & Yong. Using whole-genome re-sequencing they identified strong population structure and selective sweeps in highly differentiated regions, with candidate genes underlying local adaptation to temperature-related variables. This study provides crucial insights into how genomic variation underlies a species’ ability to adapt to specific environmental conditions (Bragg et al., 2015).

This Research Topic also showcases advanced genomic approaches that facilitate a deeper understanding of plant diversification (Soltis and Soltis, 2021). Monloy and Planta provide a comprehensive analysis of tRNA gene content, structure, and organization across the flowering plant lineage. Their comparative genomic study reveals variation in the number of nuclear tDNAs and distinct clustering patterns among different plant groups, providing a valuable foundation for future research on tRNA gene function and regulation. Furthermore, Tao et al. utilized complete chloroplast genome data to investigate the *Solidago canadensis* L. complex and its anthropogenic introduction pathways into China. Their *de novo* assembled chloroplast genomes offer important insights into phylogenetic relationships, sequence divergence, and potential introduction routes, demonstrating the power of organellar genomics in understanding invasion biology and evolutionary history (Keller and Taylor, 2008).

In summary, the articles in this Research Topic collectively underscore the pivotal role of genome and chromosome evolution in driving plant diversification (Soltis and Soltis, 2021). From ancient polyploidization events that shaped entire plant families to the subtle chromosomal rearrangements influencing local adaptation and speciation at microevolutionary levels, the contributions highlight the dynamic and intricate mechanisms at play. Furthermore, the interplay between these genomic changes, reproductive strategies, and environmental correlates provides a more complete scenario of how plants adapt and diversify (Cowling and Pressey, 2001). The ongoing advancements in genomic technologies are clearly helping researchers to unravel these complex processes with unprecedented detail (Zuntini et al., 2024). We hope this Research Topic serves as a valuable resource for researchers interested in the genetic and chromosomal underpinnings of plant evolution and inspires further investigations into these fascinating areas.
